# Functional characterization of the human tRNA methyltransferases TRMT10A and TRMT10B

**DOI:** 10.1093/nar/gkaa353

**Published:** 2020-05-11

**Authors:** Elisa Vilardo, Fabian Amman, Ursula Toth, Annika Kotter, Mark Helm, Walter Rossmanith

**Affiliations:** 1 Center for Anatomy & Cell Biology, Medical University of Vienna, 1090 Vienna, Austria; 2 Department of Theoretical Chemistry, University of Vienna, 1090 Vienna, Austria; 3 Institute for Pharmacy and Biochemistry, Johannes Gutenberg-University, 55128 Mainz, Germany

## Abstract

The TRM10 family of methyltransferases is responsible for the *N*^1^-methylation of purines at position 9 of tRNAs in Archaea and Eukarya. The human genome encodes three TRM10-type enzymes, of which only the mitochondrial TRMT10C was previously characterized in detail, whereas the functional significance of the two presumably nuclear enzymes TRMT10A and TRMT10B remained unexplained. Here we show that TRMT10A is m^1^G9-specific and methylates a subset of nuclear-encoded tRNAs, whilst TRMT10B is the first m^1^A9-specific tRNA methyltransferase found in eukaryotes and is responsible for the modification of a single nuclear-encoded tRNA. Furthermore, we show that the lack of G9 methylation causes a decrease in the steady-state levels of the initiator tRNA^iMet-CAT^ and an alteration in its further post-transcriptional modification. Our work finally clarifies the function of TRMT10A and TRMT10B in vivo and provides evidence that the loss of TRMT10A affects the pool of cytosolic tRNAs required for protein synthesis.

## INTRODUCTION

Although RNA modifications are known since almost 60 years, the investigation of the pathways and functions linked to RNA modification has lingered for decades, partially due to the lack of methodologies. To date, only some of the enzymatic pathways responsible for RNA modification have been identified (1), and only very few have been characterized in detail. Mutations in about a half of the currently known RNA modification enzymes are associated to human diseases, manifested with diverse phenotypes, ranging from mild metabolic disorders to severe neuropathies (2). Methylations are the most abundant type of modifications found in RNA, accounting for about two thirds of the more than 150 known modifications (reviewed in 3). Most of the reported RNA methylation sites are located in tRNAs, though many are also found in rRNAs and other coding and non-coding RNAs (4).

**Figure 1.**
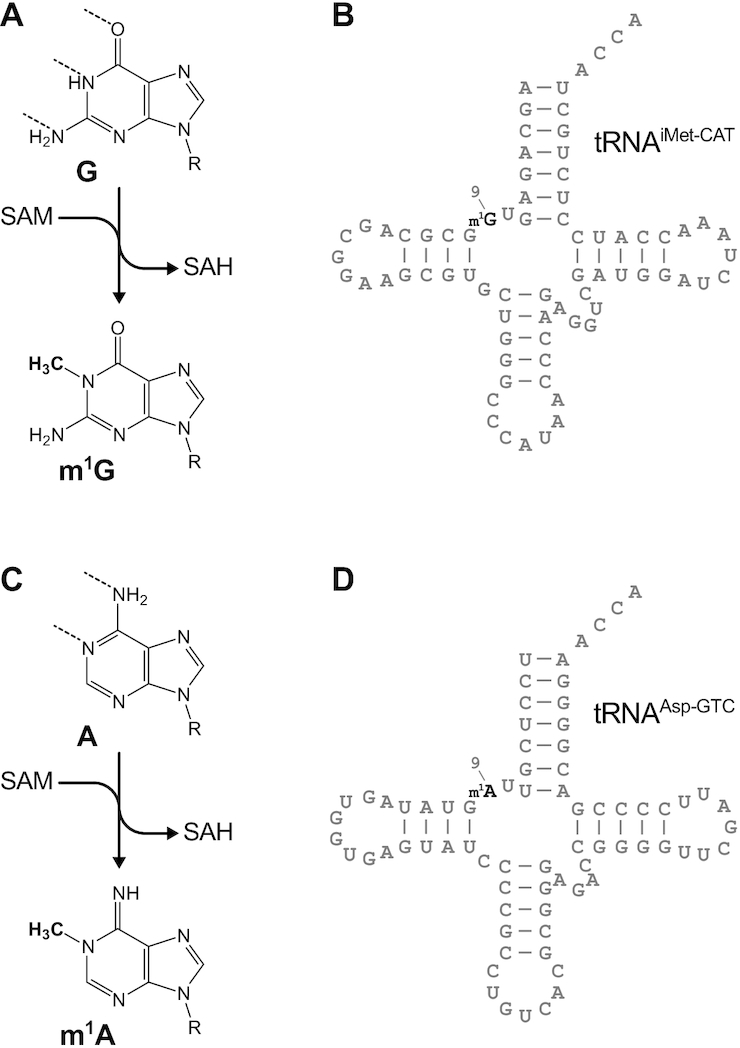
Methylation of nitrogen-1 (*N*^1^) of purines at position 9 of tRNAs. (**A**) Enzymatic methylation of guanosine using SAM as the methyl group donor and release of S-adenosyl homocysteine (SAH). (**B**) Cloverleaf structure of human tRNA^iMet-CAT^ with the modified guanosine at position 9 highlighted in bold. (**C**) Enzymatic methylation of adenosine. (**D**) Cloverleaf structure of human tRNA^Asp-GTC^ with the modified adenosine at position 9 highlighted in bold.

The methylation of nitrogen-1 (*N*^1^) of purines at position 9 of tRNAs (m^1^R9) is a modification located in the core of tRNAs (Figure 1), believed to be important for folding and structural stability (5,6). Although widely conserved in Archaea, and in nuclear and mitochondrial tRNAs of Eukarya, it was shown to be non-essential in yeast, and to cause a growth phenotype only in combination with additional modification deficiencies or under stress (7). The enzyme family responsible for the methylation of position 9 is the TRM10 family, whose eponym, yeast Trm10p, was the first member identified (8). In humans and other vertebrates, the TRM10 family is expanded to three enzymes, namely TRMT10A, TRMT10B, and TRMT10C. We originally identified the TRMT10C protein as a component of the human mitochondrial RNase P complex (9), and later we showed that TRMT10C together with the partner protein SDR5C1 is a dual-specificity methyltransferase, responsible for the methylation of A or G at position 9 of mitochondrial tRNAs (10). We and others also showed that TRMT10A is an active tRNA G9 methyltransferase *in vitro*, suggesting that it may be responsible for the methylation of nucleo-cytoplasmic tRNAs (10–13). We previously found TRMT10B to be inactive on several tested tRNA substrates (we recently revised an erroneously reported G9 methyltransferase activity of TRMT10B) (10,14). However, during the revision of this manuscript, others reported recombinant TRMT10B to be able to weakly methylate *in vitro* the A9 of tRNA^Asp-GTC^ and, with even lower activity, also the G9 of two further tRNAs (13). Thus, the actual enzymatic specificity of TRMT10B and its function *in vivo* remain to be elucidated. Remarkably, mutations in the gene of TRMT10A were recently reported to be associated with a severe medical condition characterised by microcephaly and early onset diabetes (15–19). These findings highlight the need to better understand the function of the members of the human TRM10 enzyme family and their impact on cellular physiology.

In the present work, we sought to identify the targets of TRMT10A and TRMT10B in human cells, to discern their possible functional redundancy/specificity, and to ultimately shed light on the importance of position-9 methylation in nucleocytoplasmic tRNAs. We show that TRMT10A and TRMT10B are both tRNA:m^1^R9 methyltransferases, yet with a unique, non-overlapping set of tRNA targets, and different purine specificity: TRMT10A is guanosine-specific, whereas TRMT10B is the first identified adenosine-specific member of the TRM10 family in Eukarya. We also show that the lack of position-9 methylation causes a decrease in the steady-state levels and an alteration in the modification profile of at least one of the affected (normally methylated) tRNAs, pointing to a role of the modification in tRNA stability and/or turnover.

## MATERIALS AND METHODS

### Knock out cell lines generation and analysis

Wild type haploid HAP1 cells, two KO clones for *TRMT10A* and two KO clones for *TRMT10B* were obtained from a commercial source (Horizon) and cultivated in adhesion in IMDM medium supplemented with 10% foetal calf serum.

Double KO cell lines were generated by CRISPR/Cas mediated genome editing (20). The vector pX330-U6-Chimeric_BB-CBh-hSpCas9 encoding *S. pyogenes* Cas9 and the chimeric guide RNA scaffold was obtained from Addgene (#42230) and modified: a cassette containing a CMV promoter followed by the coding sequence of eGFP and SV40 polyA site was inserted into its *Esp*3I/*Not*I sites. This plasmid, renamed pX330g, was used for cloning a guide RNA targeting the sequence AACTCAGATGGACATGAC in exon 3 of *TRMT10A* into the chimeric guide RNA scaffold. Another guide RNA targeting the sequence GATCACTTGATGGTATTAA in exon 3 of *TRMT10A* was cloned in the vector pU6, encoding the human U6 promoter followed by the chimeric guide RNA scaffold, and U6 terminator. The two plasmids were co-electroporated into HAP1 *TRMT10B*-KO clone #4 (ΔB4), and single-cell clones were isolated by limiting dilution. Clones were expanded, genomic DNA was extracted, and the clones were screened for deletions by PCR using the primers CTGAAGCTTTTAGATGGGTT and ACAAAAATGATTCGACCTAATTCAC. The PCR amplicon of two selected positive clones was gel-purified and verified by Sanger sequencing.

The expression of the TRMT10A protein was verified by western blotting. Briefly, cells were broken in lysis buffer (60 mM Tris·Cl pH 6.8, 0.5 mM DTT, 2% SDS, 10% glycerol), proteins separated by Tris·glycine SDS-PAGE, electro-blotted on PVDF membrane, and probed with anti-TRMT10A (SAB2700685, 1:1000, rabbit, Sigma) or anti-GAPDH (6C5, 1:2000, mouse, Ambion) antibodies overnight at 4°C. The membrane was then developed with secondary, peroxidase-coupled anti-rabbit or anti-mouse antibodies, followed by chemiluminescence imaging.

Commercially available and custom-made anti-TRMT10B antibodies did not perform satisfactorily, and TRMT10B could not be analysed by western blotting. Thus the *TRMT10B* KO was tested at the RNA level by RT-PCR. Total RNA was extracted with Ribozol (Amresco), and cDNA was synthesized using oligo-dT primers. A cDNA fragment encompassing the CRISPR target sites was amplified by PCR with primers TTGGAAACCCTTGTGTACCTG and GGCAAGCGTGCGGTCTTGACA.

### Cell proliferation assay

Real-time cell proliferation assays were performed on an xCELLigence^®^ RTCA DP system (ACEA Biosciences). Cells were seeded in the E-plates, 15000 cells/well, each cell line in quadruplicate, and incubated in IMDM medium supplemented with 10% foetal calf serum. Real-time resistance-measurement data were recorded and presented as arbitrary units called cell index. The change in cell index was recorded for 96 hours, and used to calculate the cell-index doubling time with the provided RTCA analysis software (ACEA Biosciences). The data were fitted to the exponential equation CI_*i*_ = CI_1_*2^(ti-t1)/DT^, were *i* = 1, 2, 3, …, n, DT is the cell-index doubling time, and CI_*i*_ is the cell index at time point *t_i_*. The analysis was restricted to the time window of exponential growth.

### Next generation sequencing and analysis for modification detection

Total RNA of wild type and KO HAP1 cell lines was extracted with RiboZol (Amresco), separated by denaturing PAGE, and the small RNA fraction (∼50–150 nt) containing tRNAs and precursors was eluted from the gel. The RNA was subjected to pyrophosphate hydrolysis with RppH (New England Biolabs) at 37°C for 30 min to convert the 5′ triphosphate of pre-tRNAs to 5′ monophosphate; subsequently, the RNA was deacylated in 200 mM Tris·Cl pH 9.5 at 37°C for 2 h (21), and re-purified by phenol chloroform extraction. 50 ng of each RNA sample was further processed for library preparation using the NEBNext Multiplex Small RNA Library Prep Set for Illumina (New England Biolabs) according to manufacturer's protocol. The libraries were sequenced (single-read 100 nt) on an Illumina HiSeqV4 by the Vienna BioCenter Core Facilities’ NGS service. A total of nine libraries was generated from three independently prepared RNA samples of wild type cells, and one RNA sample of each KO clone.

Obtained RNA-seq reads were subject to standard quality control. Adapter and low-quality bases were removed using BBduk (https://github.com/BioInfoTools/BBMap), quality was monitored before and after cleansing using FastQC (http://www.bioinformatics.babraham.ac.uk/projects/fastqc/). RNA base modifications were detected as recently described (22). Briefly, reads were separated into precursor-tRNA derived reads by constructing a reference sequence library containing the human reference genome hg38 with masked tRNA loci with additionally attached sequences representing tRNA sequences with each 50 nt flanking region. The tRNA coordinates were obtained scanning the reference genome using tRNAscan (23) once with the default settings and once with the adapted tRNA model to detect mitochondrial tRNAs. Mitochondrial tRNA^Ser-AGY^ was missing from this in-silico prediction, and was manually added based on information obtained from mitotRNAdb (24). The reads were aligned against these constructed reference sequences. Reads mapping to the core reference genome or (partially) to the tRNA gene flanking regions were eliminated. The remaining reads were mapped to a reference sequence library consisting of mature tRNA sequences (introns removed, CCA tails added), whereby identical tRNAs were collapsed into one representative sequence. For both mapping steps, the short-read aligner segemehl was used with relaxed stringency parameter (25). The read alignments were further realigned with GATK’s (Genome Analysis Toolkit) IndelRealigner (26) to minimize the number of mismatches across all reads for a given locus. Eventually, multimapper, i.e. reads which map to multiple loci in the reference genome with equal alignment scores, were filtered by allowing only uniquely mapped reads, or reads which map to all their mapping sites not just equally well but with identical alignments. To increase sensitivity in modification site calling we deviated from the previously reported best practice workflow (22) and we used the tailor-made approach ‘pfropfen’ to call sites with increased mis-incorporation rates for all possible base substitution events. Each individual substitution rate was tested whether it was significantly greater than the observed background occurrence for this type of substitution. The obtained *P*-values were winsorized (removing the highest and lowest *P*-value) and merged applying Fisher's method, resulting in an overall *P*-value for each site, which is eventually corrected for multiple testing applying the method of Benjamini & Hochberg. Sites with coverage of at least 10 reads, and a FDR < 0.01 in at least one of the examined samples were considered significant and processed for further analysis. The source code is available at https://github.com/fabou-uobaf/Helferlein/blob/master/Pfropfen. For each potential modification site detected in any of the analysed samples, the mis-incorporation rates for all samples were deduced using the software bam-readcount with parameter –min-base-quality and –min-mapping-quality set to 20.

### RNA-SCRATCh methylation assay

About 300 μg of total RNA, or the gel-purified tRNA fraction derived from 250 μg of RNA, were annealed to a chimeric ssODN complementary to 18 nt of the assayed tRNA. The sequences of these ssODNs were the following, where Nm stands for 2′-*O*-methyl-*N*: anti-tRNA^iMet-CAT^ G9 CmCmGmCmUmGmCmGmCmCmACTCUmGmCmUm, anti-tRNA^iMet-CAT^ G10 CmCmGmCmUmGmCmGmCmCACTCmUmGmCmUm, anti-tRNA^iMet-CAT^ G26 GmCmCmCmAmGmCmACGCTmTmCmCmGmCmTm, anti-tRNA^Asp-GTC^ G9 CmCmAmCmUmAmUmAmCmUmAACGAmGmGmAm, anti-tRNA^Pro-AGG^ G9 CmCmCmCmUmAmGmAmCmCmAACGAmGmCmCm. 5 units of FastAP (Thermo Scientific) and 15 units of RNase H (Epicenter) were added and the reactions were incubated at 37°C for 15 min, followed by inactivation at 65°C for 20 min. The cleaved tRNA 3′ fragment was isolated by annealing to a complementary biotinylated ssODN and pull down with MagneSphere^®^ Streptavidin beads (Promega). The sequences of the ssODNs employed were the following (-bio stands for biotin attached via a triethylene glycol spacer): anti-tRNA^iMet-CAT^ TTTCGATCCATCGACCTCTGGGTTATGGG-bio, anti-tRNA^Asp-GTC^ AATCGAACCCCGGTCTCCCGCGTGACAGG-bio, anti-tRNA^Pro-AGG^ ATTTGAACCCGGGACCTCTCGCACCCTAA-bio. The capture probes and conditions used are not expected to be selective among closely related isoforms of the respective tRNA. The RNA eluted from the beads was phenol-chloroform extracted and precipitated, ^32^P labeled at the 5′ end with T4 polynucleotide kinase, and purified by denaturing PAGE. The isolated, labeled tRNA fragment was further processed for one-dimensional or 2D thin layer chromatography (TLC) as previously described (10,27).

### Expression and purification of recombinant proteins

The plasmids for the expression of N-terminally His-tagged TRMT10A, TRMT10B, SDR5C1 and Trm10p, and native (untagged) TRMT10C, were previously described (8–10). The protein preparations of Trm10p and the TRMT10C-SDR5C1 complex were previously described (10). TRMT10A and TRMT10B were expressed in *E. coli* BL21(DE3) and purified as previously described with minor changes (10): bacteria were lysed by sonication and the two proteins were purified on His SpinTrap columns, or on HisTrap HP columns using an ÄKTApurifier chromatography system (GE Healthcare). Bacterial lysates in buffer A (150 mM NaCl, 50 mM HEPES·Na pH 7.4, 10% glycerol, 1 mM DTT, 0.2% Tween, 0.2% Protease Inhibitor) and 50 mM imidazole were loaded on column, washed with loading buffer, washed with 1 M NaCl in buffer A, washed with buffer A, then with 150 mM imidazole in buffer A, and eluted with 500 mM imidazole in buffer A. The purity and concentration estimation of all recombinant proteins relative to BSA standards was assessed by SDS-PAGE followed by Coomassie brilliant blue staining. Purified proteins were snap-frozen in liquid nitrogen and stored in aliquots at −80°C. Both TRMT10A and TRMT10B were purified multiple independent times and gave consistent results with respect to activity and specificity.

### Preparation of tRNA substrates

The template plasmid for human tRNA^iMet-CAT-2^ encoding T7 promoter, hammerhead ribozyme, and nucleotides 9–72 of the tRNA followed by CCA was generated by overlap extension PCR and cloned in pGEM-1 (Promega). The templates for human tRNA^Asp-GTC-2^ and the variant tRNA^Asp-GTC-A9G^ were generated by gene synthesis (BIOMATIK), and encoded T7 promoter, hammerhead ribozyme, nucleotides 9–72 of the tRNA followed by CCA, and a 3′ HDV ribozyme. The tRNA^iMet-CAT-2^ and the two tRNA^Asp-GTC^ templates were cleaved with BstNI and HindIII, respectively, and the tRNA fragments were produced as run-off transcripts guided by T7 RNA polymerase. The *in vitro* transcribed tRNA fragments were 5′-end labelled and ligated by splint-guided ligation to a synthetic RNA corresponding to the first eight nucleotides of the respective tRNA. *In vitro* transcription, ^32^P labeling at position 9, purification, and splint-guided ligation were carried out as previously described (10).

### Methyltransferase assay

Methylation activities were assayed under single turnover conditions at 30°C in methylation buffer (50 mM Tris·Cl pH 8, 20 mM NaCl, 4.5 mM MgCl_2_, 2 mM DTT, 20 μg/ml BSA, 0.5 units/μl Ribolock RNase inhibitor (Thermo Scientific)), in the presence of 25 μM *S*-adenosyl methionine (SAM) and position-9 labeled tRNA substrate (<10 nM). Recombinant Trm10p, TRMT10C-SDR5C1 complex, TRMT10A and TRMT10B were added to a final concentration of 250 nM, and incubated for 3 h. Reactions were stopped by adding 125 mM guanidine hydrochloride, and further processed for TLC as previously described (10).

### Northern blotting

5 μg of total RNA extracted from HAP1 wild type and knock out cell lines were separated by denaturing PAGE, electro-blotted onto Hybond-N Nylon membrane (GE Healthcare) and UV-crosslinked. The membrane was incubated in blocking solution (6× SSC, 10× Denhardt's solution, 0.5% SDS, 0.1 mg/ml salmon sperm) and subsequently probed with a complementary ssODN ^32^P labeled at the 5′ end with T4 polynucleotide kinase. The probes and hybridization temperatures used were the following: tRNA^iMet-CAT^ TAGCAGAGGATGGTT (37°C), tRNA^Asp-GTC^ CTCCCCGTCGGGGAA (37°C), 3′ tRNA^Gln-UUG/CUG^ AGGTCCCACCGAGAT (37°C), 5′ tRNA^Gln-UUG/CUG^ GTGCTAACCATTACACCATGG (42°C), 5S rRNA AAAGCCTACAGCACCCGGTATT (42°C). Of notice, the tRNA probes used were short and targeted to the 3′ end of the respective tRNA, thus unable to distinguish among closely related tRNA isoforms. The only exception was the probe targeted toward the 5′ of tRNA^Gln-UUG/CUG^, as used in (12). Washed membranes were subjected to storage phosphor autoradiography and the detected bands were quantitated by densitometry. The signals of tRNA^iMet-CAT^, tRNA^Asp-GTC^ and tRNA^Gln-UUG/CUG^ were normalized to 5S rRNA, and the data of three independent replicates for tRNA^iMet-CAT^ and tRNA^Gln-UUG/CUG^, and four replicates for tRNA^Asp-GTC^ were collected and plotted relative to wild type HAP1. The steady-state levels of the tested tRNAs in KO versus wild type cell lines were compared by one-way ANOVA followed by Dunnett's multiple comparison test, using Prism 5 (GraphPad Software). *P*-values < 0.05 were considered significant.

### tRNA^iMet-CAT^ isolation by affinity chromatography

An ssODN complementary to tRNA^iMet-CAT^ (sequence TAGCAGAGGATGGTTTCGATCCATCGT) was covalently bound via a 5′ amino-linker to a HiTrap^TM^ NHS-activated HP chromatographic column according to the manufacturer's instructions (GE Healthcare). tRNA^iMet-CAT^ was isolated as previously described (28) with minor changes. Briefly, the small (<200 nt) RNA fraction was isolated from wild type or ΔA4 HAP1 cells using RNAzol (Molecular Research Center), diluted in binding buffer (0.5 M NaCl, 50 mM Tris–Cl pH 7.1, 15 mM EDTA) and circulated on the column at 60°C for 4 h with the aid of a peristaltic pump. The column was subsequently washed with 10 column volumes of wash buffer (50 mM NaCl, 5 mM Tri-Cl pH 7.1, 1 mM EDTA) at 40°C, and the RNA was eluted with 2 column volumes of H_2_O at 75°C for 3 min, and precipitated with isopropanol and NH_4_OAc at −20°C.

### Mass spectrometry

The isolated tRNA^iMet-CAT^ WT and KO samples were digested into nucleosides using 0.6 U nuclease P1 from *P. citrinum* (Sigma-Aldrich), 0.2 U snake venom phosphodiesterase from *C. adamanteus* (Worthington), 2 U FastAP (Thermo Scientific), 10 U benzonase (Sigma-Aldrich) and 200 ng pentostatin (Sigma-Aldrich) in 25 mM ammonium acetate (pH 7.5; Sigma-Aldrich) over night at 37°C. The nucleosides were then spiked with internal standard (^13^C stable isotope-labeled nucleosides from *S. cerevisiae*, SIL-IS) and subjected to analysis. Three independent replicates of the ΔA4 samples and three of the WT samples (with the exception of dihydrouridine, which could only be analysed in two WT samples) with 50 ng internal standard were analysed via LC-MS (Agilent 1260 series and Agilent 6460 Triple Quadrupole mass spectrometer equipped with an electrospray ion source (ESI)). The solvents consisted of 5 mM ammonium acetate buffer (pH 5.3; solvent A) and LC–MS grade acetonitrile (solvent B; Honeywell). The elution started with 100% solvent A with a flow rate of 0.35 ml/min, followed by a linear gradient to 8% solvent B at 10 min and 40% solvent B after 20 min. Initial conditions were regenerated with 100% solvent A for 10 min. The column used was a Synergi Fusion (4 μM particle size, 80 Å pore size, 250 × 2.0 mm; Phenomenex). The UV signal at 254 nm was recorded via a diode array detector (DAD) to monitor the main nucleosides. ESI parameters were as follows: gas temperature 350°C, gas flow 8 l/min, nebulizer pressure 50 psi, sheath gas temperature 350°C, sheath gas flow 12 l/min, capillary voltage 3000 V. The MS was operated in the positive ion mode using Agilent MassHunter software in the dynamic MRM (multiple reaction monitoring) mode. For relative quantification, an internal calibration was applied as described previously (29).

### Global protein synthesis assay

The level of global protein translation was assayed in HAP1 wild type and KO clones using a Global Protein Synthesis assay kit (Abcam) according to manufacturer's protocol. Briefly, cells seeded in 96-well cluster plate were treated with O-Propargyl-puromycin (OP-puro) added directly in the culture medium; the cells were then resuspended, fixed, permeabilized, and labelled via click chemistry with 5-TAMRA. Labelled cells were subsequently analysed on a CytoFLEX flow cytometer (Beckman Coulter). The median cell fluorescence intensity was calculated per sample; a total of six replicates were compared and plotted as means, relative to wild type.

## RESULTS

### TRMT10A and TRMT10B are not essential in human cells

To investigate the function of TRMT10A and TRMT10B, and clarify their possible redundancy, we used human knockout (KO) cell lines obtained by CRISPR/Cas genome editing. Two KO clones for *TRMT10A* (here abbreviated ΔA4 and ΔA10) and two for *TRMT10B* (ΔB4 and ΔB9) were generated by a commercial supplier in HAP1 cells, a nearly-haploid human cell line derived from the chronic myelogenous leukemia cell line KBM-7. In addition, we targeted *TRMT10A* in clone ΔB4, and isolated two double KO clones for further experiments (ΔAB2 and ΔAB4). The genotype of the obtained clones was verified by genomic DNA amplification and Sanger sequencing (Table 1; Supplementary Figure S1A), and further validated by western blotting for TRMT10A and reverse transcription-PCR (RT-PCR) for TRMT10B (Supplementary Figure S1B and C). All but one KO clones used contained mutations causing a frameshift and early stop deleting the entirety of the catalytic domain (Table 1); in the only exception ΔAB2, the 32-amino acids in-frame deletion removed part of the N-terminus of TRMT10A and the central β-strand of its catalytic domain, and is thus expected to be detrimental for folding and methyltransferase activity.

**Table 1.** Genotype of single and double KO clones used in the study

Target gene	Clone	Mutation	Effect on protein
*TRMT10A*	ΔA4	c.224_251del (exon 3)	frameshift > stop after 28 amino acids
*TRMT10A*	ΔA10	c.250_251insT (exon 3)	frameshift > stop after 4 amino acids
*TRMT10B*	ΔB4	c.688_689insCA (exon 7)	frameshift > stop after 13 amino acids
*TRMT10B*	ΔB9	c.665_696del (exon 7)	frameshift > stop after 3 amino acids
*TRMT10A*/*TRMT10B*	ΔAB2	c.250_342delinsCT (exon 3)/ c.688_689insCA (exon 7)	frameshift > stop after 1 amino acid/ frameshift > stop after 13 amino acids
*TRMT10A*/*TRMT10B*	ΔAB4	c.251_319del (exon 3)/ c.688_689insCA (exon 7)	in frame deletion of 23 amino acids/ frameshift > stop after 13 amino acids

The genotype of the different cell lines was verified by genomic DNA amplification and subsequent Sanger sequencing of the regions targeted in CRISPR/Cas genome editing. The numbering refers to the nucleotide position in the mRNA coding sequence starting from the translation start, according to the Human Genome Variation Society's guidelines (44).

**Figure 2.**
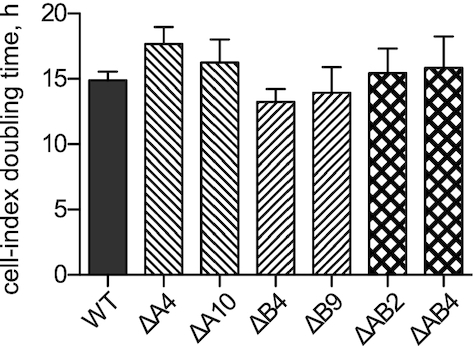
Growth of HAP1 wild type (WT), TRMT10A KO, TRMT10B KO and double KO clones. Cell growth was monitored by a real-time cell proliferation assay, and the change in cell index was used to calculate the cell-index doubling time. The results are shown as mean and SD of multiple independent replicates (*N* = 5 for WT, ΔA4, ΔA10, ΔAB4; *N* = 3 for ΔB4, ΔB9, ΔAB2).

Single and double KO clones did not display any obvious phenotype. We used a real-time cell proliferation assay to measure the apparent doubling time of the different KO clones in comparison to the parental wild type HAP1 cell line. The method measures the electrical impedance, and the changes therein, of cells growing in adhesion on the electrode-coated wells of a microtiter plate. The resulting changes in conductivity are inversely correlated to the surface of the well covered by the cells’ monolayer, and is used to calculate the approximate growth rate of the cell line. The obtained apparent doubling time of the different lines was not significantly different compared to wild type cells (∼15 h), although we observed a trend to slower growth of ΔA4 and ΔA10 compared to wild type, and of ΔAB2 and ΔAB4 compared to the parental line ΔB4 (Figure 2).

### TRMT10A and TRMT10B methylate different nuclear-encoded tRNAs and have distinct purine specificities

**Figure 3.**
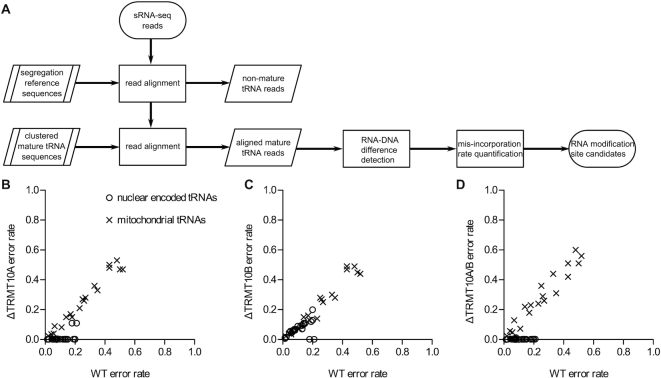
tRNA NGS and detection of methylation at position 9. (**A**) Workflow to detect RNA modification on the basis of misincorporation marks in NGS data. The quality-controlled sRNA reads were first aligned to a reference genome consisting of the human genome with masked tRNA loci and attached sequences of tRNA including 50 nt flanking regions. Reads mapping either to the core genome or to the flanking regions were removed. Subsequently, the remaining reads were aligned to a reference sequence consisting of mature tRNA sequences, whereby tRNAs of identical sequence are collapsed into one representative. From there, alignment positions with significantly more RNA–DNA differences than expected by background sequencing errors were identified. For these candidate tRNA positions, the rate for each base substitution was quantified and reported. (**B**) Error rates at position 9 of tRNAs that were identified as modified, were compared between wild type HAP1 (WT) and TRMT10A KO (ΔTRMT10A) cells. Data are shown as the mean error rate of the three sequencing replicates of wild type cells and of the two TRMT10A KO clones. (**C**) Error rates at position 9 were compared like in (B) between wild type HAP1 cells (WT; *N* = 3) and the TRMT10B KO clones (ΔTRMT10B, *N* = 2). (**D**) Error rates at position 9 were compared like in (B) between wild type HAP1 cells (WT; *N* = 3) and TRMT10A/TRMT10B double KO clones (ΔTRMT10A/B, *N* = 2).

The *N*^1^ methylated by the TRM10 enzymes is located at the Watson–Crick edge of the purine (Figure 1A and C), and its modification interferes with canonical base pairing, causing errors during reverse transcription (polymerization abortion and/or random nucleotide incorporation). This interference with the reverse transcriptase (RT) has been long used to assay RNA methylation (30). More recently, the combination with next generation sequencing (NGS) allowed to use the resulting RT-error signature to detect *bona fide* RNA methylation sites at the transcriptomic level (reviewed in 31). Here we used a similar NGS-based strategy to identify all nuclear encoded tRNAs that are methylated at position 9 in HAP1 cells, and to study whether their modification is affected by the knockout of *TRMT10A* and/or *TRMT10B*. We isolated cellular small RNA in the range 50–150 nt to include the tRNAs as well as their unprocessed precursors, and aligned the obtained reads according to a mapping strategy that we previously developed specifically for tRNA sequencing (22) (Figure 3A). We successfully detected an error signature significantly above background indicative of modification at position 9 in 12 nuclear encoded tRNAs, and in all 19 mitochondrial tRNAs that have a purine at this site (Table 2 and Supplementary Table S1). We also observed this error signature in six additional tRNAs, which were previously reported to contain m^1^G9 (32), but did not pass our stringent initial inclusion criteria. Upon KO of *TRMT10A*, the modification signature at position 9 was lost in 17 out of the 18 nuclear-encoded tRNAs (including the six tRNAs identified with lower confidence), whereas upon KO of *TRMT10B* only tRNA^Asp-GTC^ lost the modification (Figure 3B and C; Table 2). Remarkably, all the 17 tRNAs selectively affected in the *TRMT10A* KO contain a guanosine at position 9, whereas tRNA^Asp-GTC^ has an adenosine at position 9, and was recently reported as the first and probably only case of m^1^A9 in human nuclear-encoded tRNAs (32). In the double KO cells, all 18 nuclear-encoded tRNAs that are modified in HAP1 lost the modification signature (Figure 3D). The modification signature was only very low in reads extending beyond the 5′ and/or 3′ end of mature tRNAs (data not shown), indicating that the methylation at position 9 takes place mostly after 5′- and 3′-end processing of tRNA precursors. Finally, as expected, neither the knockout of *TRMT10A* nor that of *TRMT10B* affected the modification of any mitochondrial tRNA (Figure 3B–D), which were previously shown to be methylated by the TRMT10C–SDR5C1 complex (10).

**Table 2.** Nuclear-encoded tRNAs modified at position 9

			Mean error rate			
tRNA	Purine 9	GtRNAdb ID	WT	ΔA	ΔB	ΔAB
Arg^CCG^	G	tRNA-Arg-CCG-2	0.14	0.0024	0.11	0.0019
		NA	0.067	0.0032	0.059	0.0053
Arg^CCT^	G	tRNA-Arg-CCT-2	0.079	0.001	0.066	0.0052
		tRNA-Arg-CCT-1	0.076	0.001	0.064	0.0056
		tRNA-Arg-CCT-4	0.014	0.0013	0.011	0.00082
		tRNA-Arg-CCT-3	0.044	0.003	0.047	0.0033
Arg^TCT^	G	tRNA-Arg-TCT-2	0.2	0.0018	0.13	0.0028
		tRNA-Arg-TCT-5	0.2	0.0017	0.13	0.0029
		tRNA-Arg-TCT-3	0.19	0.0022	0.12	0.0028
		tRNA-Arg-TCT-1	0.082	0.0049	0.062	0.012
Gln^CTG^	G	tRNA-Gln-CTG-2	0.082	0.0026	0.068	0.00069
		tRNA-Gln-CTG-1	0.079	0.0024	0.066	0.0012
		tRNA-Gln-CTG-5	0.073	0.0026	0.064	0.00042
Gln^TTG^	G	tRNA-Gln-TTG-4	0.051	0.0017	0.057	0.0025
		tRNA-Gln-TTG-2	0.077	0.0027	0.068	0.0025
		tRNA-Gln-TTG-3	0.066	0.0022	0.053	0.0015
		tRNA-Gln-TTG-1	0.06	0.002	0.045	0.0016
Glu^TTC^	G	tRNA-Glu-TTC-1	0.024	0.0012	0.016	0.0014
		tRNA-Glu-TTC-2	0.015	0.0013	0.0073	0.00094
iMet^CAT^	G	tRNA-iMet-CAT-2	0.2	0.0028	0.2	0.0017
		tRNA-iMet-CAT-1	0.1	0.0025	0.089	0.00085
Pro^AGG^	G	tRNA-Pro-AGG-1	0.15	0.0016	0.11	0.0012
		tRNA-Pro-AGG-2	0.13	0.0015	0.094	0.0014
Pro^CGG^	G	tRNA-Pro-CGG-2	0.15	0.0015	0.11	0.0012
		tRNA-Pro-CGG-1	0.14	0.0015	0.1	0.0012
Pro^TGG^	G	tRNA-Pro-TGG-3	0.13	0.0016	0.095	0.0012
		tRNA-Pro-TGG-2	0.12	0.0015	0.093	0.0013
Thr^CGT^	G	tRNA-Thr-CGT-2	0.13	0.0028	0.07	0.0022
		tRNA-Thr-CGT-4	0.13	0.0028	0.071	0.0022
Asp^GTC^	A	tRNA-Asp-GTC-2	0.21	0.11	0.002	0.0026
		tRNA-Asp-GTC-1	0.18	0.11	0.0016	0.0012
*Arg^ACG^	G	tRNA-Arg-ACG-1	0.026	0.0038	0.012	0.0044
		tRNA-Arg-ACG-2	0.016	0.0027	0.008	0.0034
*Arg^TCG^	G	tRNA-Arg-TCG-1	0.012	0.0013	0.012	0.0012
		tRNA-Arg-TCG-2	0.016	0.0023	0.021	0.0028
		tRNA-Arg-TCG-3	0.021	0.0025	0.017	0.0016
		tRNA-Arg-TCG-5	0.011	0.0024	0.014	0.0018
*Asn^GTT^	G	tRNA-Asn-GTT-3	0.01	0.0016	0.0039	0.0012
*Glu^CTC^	G	tRNA-Glu-CTC-1	0.0035	0.0007	0.0011	0.0005
*Thr^AGT^	G	tRNA-Thr-AGT-1	0.034	0.0045	0.014	0.0068
		tRNA-Thr-AGT-5	0.073	0.0022	0.059	0.0087
*Trp^CCA^	G	tRNA-Trp-CCA-1	0.01	0.0047	0.012	0.0018
		tRNA-Trp-CCA-3	0.016	0.005	0.017	0.0019
		tRNA-Trp-CCA-4	0.011	0.0037	0.011	0.0016
		tRNA-Trp-CCA-5	0.044	0.0034	0.02	0.0017

The error rate is reported as mean of the error observed at position 9 of the indicated tRNA in three independent replicates of wild type HAP1 tRNA sequencing (*N* = 3), and of the two biological replicates, i.e. two independent clones, for each KO condition (*N* = 2). Individual values and additional information available in Supplementary Table S1.

NA: not available in GtRNAdb at the time of analysis.

*tRNAs with low confidence modification, due to low error rate and/or read coverage.

**Figure 4.**
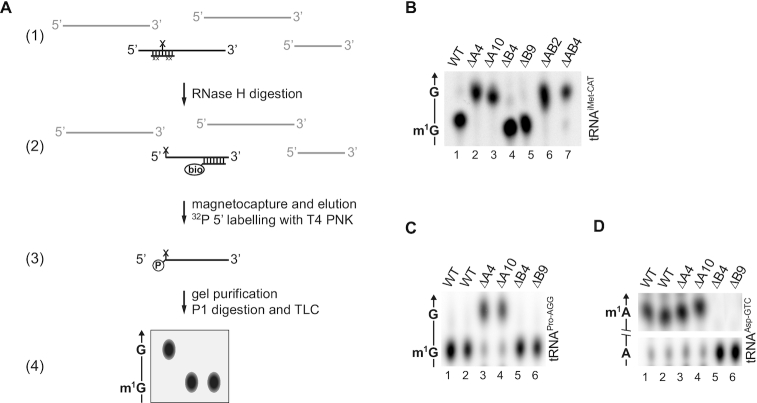
Analysis of position 9 methylation by RNA-SCRATCh. (**A**) Outline of the workflow split into 4 steps: 1) a reverse-complement chimeric ssODN is annealed to the tRNA of interest designed to guide RNase H to cleave immediately 5′ to the site of interest; 2) the resulting 3′ fragment is isolated via a biotinylated complementary ssODN and pull-down with streptavidin-coated magnetic beads, and subsequently ^32^P labelled at its 5′ end; 3) the labelled RNA is separated and purified by denaturing polyacrylamide gel electrophoresis (PAGE) to eliminate co-purified full-length tRNA and contaminants, and hydrolysed to 5′-monophosphate nucleosides; 4) the nucleosides are resolved by thin-layer chromatography (TLC) and visualized by autoradiography. (**B**) RNA-SCRATCH of position 9 of tRNA^iMet-CAT^ in WT HAP1 cells and KO clones. The arrow indicates the direction of migration of the solvent, and the identity of the spots is shown on the left. Only the relevant part of the chromatographic plate is shown. (**C**) RNA-SCRATCH of position 9 of tRNA^Pro-AGG^, like in (B). (**D**) RNA-SCRATCH of position 9 of tRNA^Asp-GTC^, like in (B). In all TLC plates, weak background spots corresponding to different nucleosides monophosphates and/or their modified forms are visible; they are likely due to purification contaminants or partial degradation before radioactive labelling.

To validate our observations with an independent method, we established a strategy consisting of RNA site-specific cleavage by RNase H followed by affinity capture and thin-layer chromatography (RNA-SCRATCh), which allows to analyze the modification status of a defined site in an RNA of interest. Briefly, specific endonucleolytic cleavage by RNase H is targeted to the phosphodiester bond upstream of the site of interest via a chimeric, complementary single-stranded oligodeoxynucleotide (ssODN). The resulting 3′ fragment is isolated by affinity magnetocapture via a biotinylated complementary ssODN, radioactively labelled at its 5′ end, gel purified, digested to 5′-monophosphate nucleosides, separated by thin-layer chromatography and visualized by autoradiography (Figure 4A). Unlike RT-based strategies like primer extension and NGS-error signature, which only hint to the presence of a modification at a certain site, RNA-SCRATCh allows to verify the identity and stoichiometry of a modification at a defined site in the RNA molecule. We selected two representative tRNAs among the targets of TRMT10A and the single TRMT10B target, and analysed them by RNA-SCRATCh. G9 of tRNA^iMet-CAT^ and tRNA^Pro-AGG^ was virtually fully modified to m^1^G9 in wild type cells, whereas the modification was absent in ΔA4 and ΔA10, as well as ΔAB2 and ΔAB4 cells (Figure 4B and C). Similarly, the A9 of tRNA^Asp-GTC^ appeared nearly fully modified to m^1^A9 in wild type cells, and the modification was lost in ΔB4 and ΔB9 cells (Figure 4D); the identity of m^1^A9 in tRNA^Asp-GTC^ was further verified by 2D thin-layer chromatography (Supplementary Figure S2).

Altogether, our results show that TRMT10A and TRMT10B are responsible for the methylation of nuclear-encoded tRNAs in human cells. Specifically, the two enzymes have a completely distinct set of tRNA target substrates and no functional redundancy, with TRMT10A being solely responsible for all the m^1^G9-containing tRNAs. Consistently, others recently observed that only human TRMT10A, but not TRMT10B, is able to complement the KO of the G9-methylating Trm10p in yeast (13). Finally, we demonstrated m^1^A9 in tRNA^Asp-GTC^ and identified TRMT10B as the enzyme responsible for this unique modification among nuclear-encoded tRNAs.

### Recombinant TRMT10A and TRMT10B have distinctive activities

**Figure 5.**
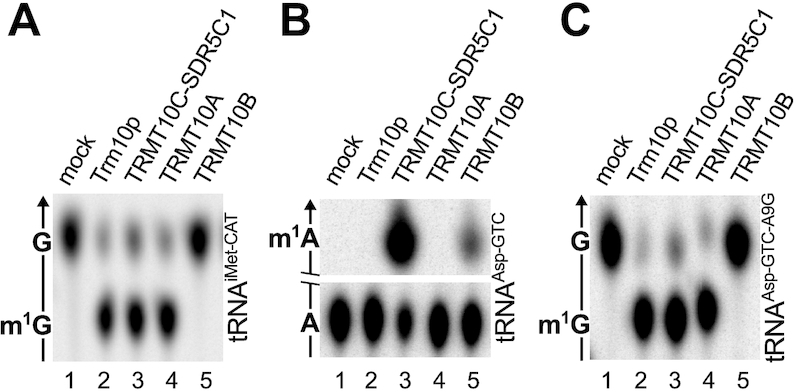
Methyltransferase activity *in vitro* of human TRMT10A and TRMT10B compared to that of other members of the TRM10 family. (**A**) Recombinant yeast Trm10p, human mitochondrial TRMT10C–SDR5C1 complex, human TRMT10A and human TRMT10B were assayed for methylation of a tRNA^iMet-CAT-2^ substrate specifically labeled at position 9; tRNA hydrolysates were resolved by TLC. The arrow indicates the direction of migration of the solvent, and the identity of the spots is labeled on the left. Only the relevant part of the chromatographic plate is shown. (**B**) The recombinant methyltransferases were assayed like in (A) with a tRNA^Asp-GTC-2^ substrate specifically labelled at position 9. (**C**) The recombinant methyltransferases were assayed like in (A) with the artificial substrate tRNA^Asp-GTC-A9G^ (A9 replaced by G) specifically labeled at position 9.

To explore the enzymatic activity of recombinant TRMT10A and TRMT10B (Supplementary Figure S3), we compared their activity *in vitro* on one of their natural tRNA substrates identified in this work. As expected, TRMT10A as well as its yeast homologue Trm10p and the human mitochondrial TRMT10C-SDR5C1 complex were able to methylate the G9 of tRNA^iMet-CAT-2^, whereas TRMT10B was completely inactive on this substrate (Figure 5A). When tRNA^Asp-GTC-2^ was used as substrate, only TRMT10B and the TRMT10C–SDR5C1 complex were able to methylate A9 (Figure 5B). Interestingly, an artificial tRNA substrate based on the sequence of tRNA^Asp-GTC-2^ where A9 was replaced by G9, was efficiently methylated by Trm10p, TRMT10A, and the TRMT10C-SDR5C1 complex, but not by TRMT10B (Figure 5C). Together with previous findings (10,11,71,14) these results confirm that TRMT10A is strictly guanosine-specific, but appears to be highly promiscuous with respect to the tRNA substrate, whereas TRMT10B seems to be able to methylate only the A9 of tRNA^Asp-GTC^, but not of any other tRNA tested, consistent with the above observed specificity *in vivo*. Moreover, our experiments confirm that TRMT10A and TRMT10B, unlike TRMT10C, are active alone and do not require additional partner proteins for activity.

### The lack of G9 methylation alters the steady state level and further modification of tRNA^iMet-CAT^

The numerous modifications in tRNAs constitute a major obstacle for RT processivity (30) and any analysis based on RT, including NGS. Thus, although the errors introduced by the RT allow the detection of modifications, they can also affect the read coverage of the tRNA itself, possibly introducing a bias, which has to be carefully considered before drawing quantitative conclusions. Indeed, we observed an increase in the read counts of most of the tRNA targets of TRMT10A and TRMT10B upon KO of the corresponding gene (Supplementary Table S1), which may be attributed to the missing RT-block, rather than to an actual increase in the level of those tRNAs.

**Figure 6.**
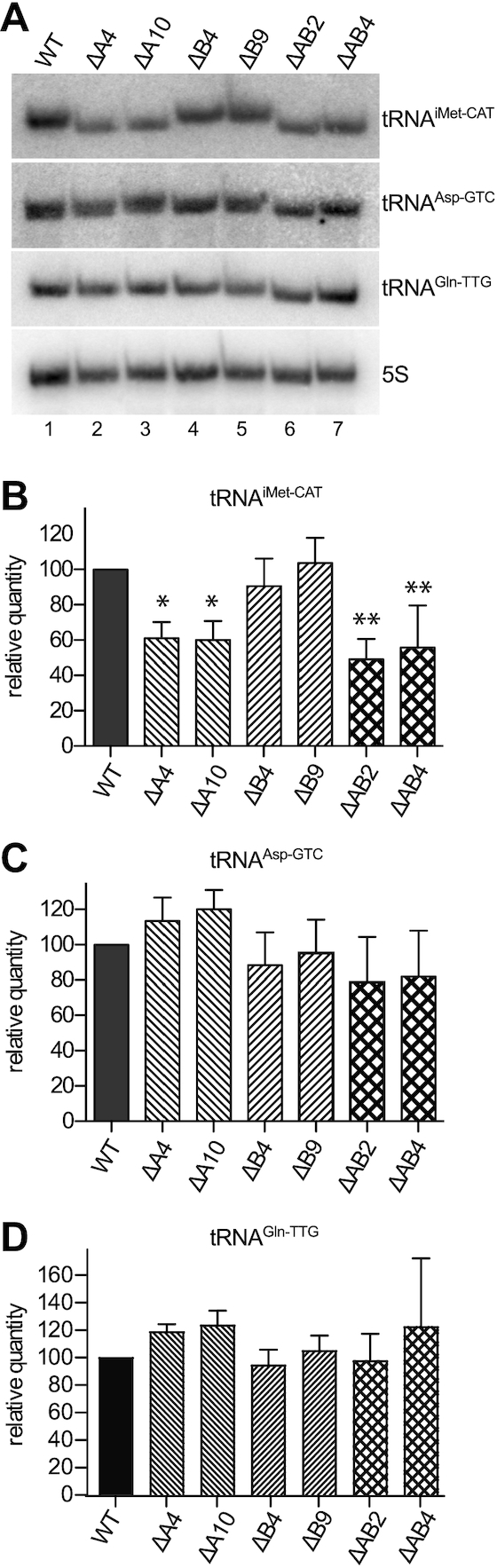
Expression levels of nuclear tRNAs upon KO of TRMT10A and TRMT10B. (**A**) The expression of tRNA^iMet-CAT^, tRNA^Asp-GTC^ and tRNA^Gln-TTG^ was analysed by northern blotting. 5S rRNA was probed as loading control. A representative blot is shown. (**B**) Quantification of tRNA^iMet-CAT^ expression from northern blots like in (A), calibrated according to 5S rRNA and normalized to WT. The data are shown as mean and SD of three independent northern blots (*N* = 3; *P < 0.05, **P < 0.01). (**C**) Quantification of tRNA^Asp-GTC^ expression from northern blots like in (A), calibrated according to 5S rRNA and normalized to WT. The data are shown as mean and SD of four independent northern blots (*N* = 4). (**D**) Quantification of tRNA^Gln-TTG^ expression from northern blots like in (A), calibrated according to 5S rRNA and normalized to WT. The data are shown as mean and SD of three independent northern blots (*N* = 3).

To clarify the effect of position-9 methylation on tRNA abundance, we analysed the steady-state levels of tRNA^iMet–CAT^, tRNA^Asp-GTC^ and tRNA^Gln-TTG^ by northern blotting. To avoid any effect due to the modification status of the tRNA on probe annealing, we used ssODN probes targeting the last 15 nt of the tRNAs, which are expected to be devoid of modifications (24,32). In the case of tRNA^iMet-CAT^, we observed a decrease of ∼40% upon KO of *TRMT10A* (Figure 6A and B); surprisingly, we also observed a change of migration pattern of this tRNA in denaturing polyacrylamide gel electrophoresis (PAGE) (Figure 6A), possibly due to an effect of the altered modification content on the conformation and/or interaction of the tRNA with buffer and gel matrix during separation. By contrast, we did not observe a significant difference in the steady-state levels of tRNA^Asp-GTC^ or tRNA^Gln-TTG^ between wild type HAP1 cells and any of the KO clones (Figure 6A, C and D). Others previously reported that the deficiency of TRMT10A causes the accumulation of 5′ fragments of tRNA^Gln^ in human lymphoblasts (12). We did not observe the accumulation of tRNA fragments in *TRMT10A-* or *TRMT10A*/B double-KO HAP1 cells, but we observed an increase in the signal of full length tRNA^Gln^ detected by a probe against the 5′ end of the tRNA, probably due to enhanced probe binding upon the lack of m^1^G9 (Supplementary Figure S4).

**Figure 7.**
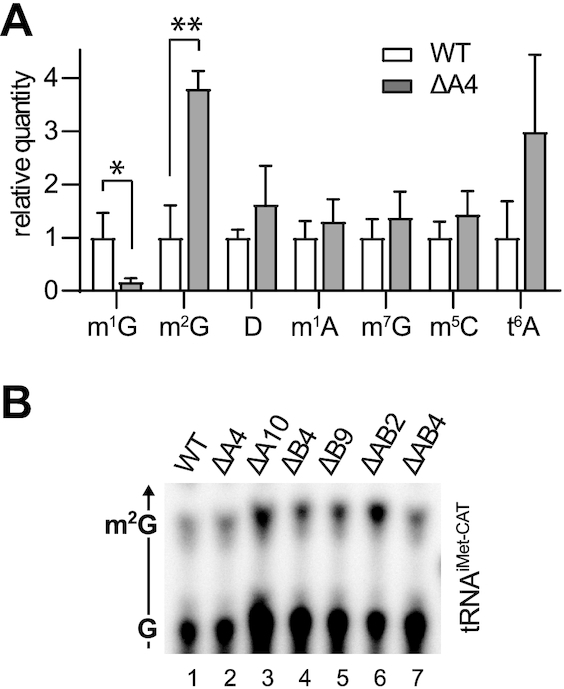
Modification profile of tRNA^iMet-CAT^. (**A**) Quantification of modified nucleosides in tRNA^iMet-CAT^ isolated from wild type or ΔA4 cells by MS. The modification abundance is expressed as relative quantity normalized to the wild type; mean and standard error are shown (*N* = 3 for all, except *N* = 2 for dihydrouridine in WT samples; *P < 0.05, **P < 0.01). (**B**) RNA-SCRATCH of position 26 of tRNA^iMet-CAT^ in WT HAP1 cells and KO clones. The arrow indicates the direction of migration of the solvent, and the identity of the spots is shown on the left. Only the relevant part of the chromatographic plate is shown.

To investigate the overall modification profile of tRNA^iMet-CAT^ upon KO of TRMT10A, we isolated this tRNA from wild type and ΔA4 HAP1 cells via affinity chromatography, and analysed it by mass spectrometry (MS). tRNA^iMet-CAT^ is characterized by a relatively low number of modifications (33), namely m^1^G9, m^2^G10, m^2^G26, t^6^A37, m^7^G46, D47, m^5^C48, and m^1^A58. All mentioned modifications were readily detectable in our MS analysis, and we confirmed that upon KO of *TRMT10A* the modification of G9 to m^1^G was abolished (Figure 7A). Interestingly, the levels of m^2^G appeared to be increased in tRNA^iMet-CAT^ isolated from ΔA4 cells, whereas we did not observe consistent change for any other modification (Figure 7A). To clarify the identity of the site of m^2^G hypermethylation we performed RNA-SCRATCh experiments targeting G10 and G26 of tRNA^iMet-CAT^. While the analysis of G10 turned out to be not possible due to a bias caused by the adjacent m^1^G9, the analysis of G26 showed no change in level of m^2^G methylation (Figure 7B), suggesting that G10 is the position hypermethylated to m^2^G upon KO of *TRMT10A*.

It was previously reported that the depletion of TRMT10A causes a slight increase of protein translation in the rat insulinoma cell line INS-1E (16). Here we measured the global protein synthesis of HAP1 cells and the derived *TRMT10A-* and/or *TRMT10B*-KO clones. Briefly, cells were treated with the puromycin derivative OP-puro, which forms covalent conjugates with nascent polypeptide chains, fluorescently labelled via click chemistry, and analysed by flow cytometry. However, as shown in Supplementary Figure S5, we did not observe any significant change in the levels of protein translation in any of our KO cell lines.

## DISCUSSION

The TRM10 family of tRNA methyltransferases is conserved in nearly all Eukarya and Archaea (34); however, little is known about their role in tRNA biology, and about the implications of the expansion of the family in vertebrates. In this work, we have shed light on the function of human TRMT10A and TRMT10B. Using an NGS-based approach, we have identified their repertoire of tRNA targets in a human cell line. Sequencing approaches have been recently used to investigate RNA modifications (reviewed in 31), and it is meanwhile evident that the specific protocol used, including the adaptor-ligation design and RT employed, can impact on the sensitivity of the method. Furthermore, the sequencing of highly repetitive elements like tRNAs poses an extra challenge by itself, since standard analysis protocols are not suited for handling reads mapping to multiple loci. Here we have used a commercially available kit for NGS library preparation that involves 5′ and 3′ adaptor ligation at the RNA level, thus it allows to further amplify and sequence only complete RT reaction products. Despite the loss of information about abortive RT reactions, the error signature that we obtained in combination with our optimized mapping strategy was sufficient to detect sites of methylation. Indeed, we detected all known m^1^R9 sites expected in mitochondrial tRNAs, corroborating the robustness of our strategy. Noteworthy, we observed good coverage, in the range of thousands of reads, for tRNA^Phe-GAA^ and tRNA^Lys-TTT^. These tRNAs are modified at position 37 to hydroxywybutosine and ms^2^t^6^A, respectively (1), which are expected to interfere with reverse transcription during library preparation. Nevertheless, we were able to detect m^1^A58 in these tRNAs (Supplementary Table S1), showing that with our approach we are able to detect modifications also in tRNAs bearing bulky, RT-stop prone modifications.

The list of nuclear-encoded tRNAs for which we detected position-9 modification is in large agreement with the tRNA methylation sites reported in a recent study in HEK293T cells (32). We found significant modification signature that was lost upon KO of *TRMT10A* in eleven tRNAs; of the additional eight tRNAs previously reported, six did not pass our stringent filtering criteria due to either a very low overall error rate, or because supported by a small number of reads. However, upon manual inspection of those sites, they showed a decrease of error rate in *TRAMT10A* and *TRMT10A/B* KO cells, suggesting that they are also methylated at G9 by TRMT10A, though possibly at very low levels. Concerning the two remaining tRNAs previously reported as methylated at position 9, we found no signature of G9 modification in tRNA^Thr-TGT^, whereas tRNA^Ile-TAT^ was not detected at all in our sequencing data. The discrepancies might be due to differences in tRNA expression (35,36) and/or modification extent between different cell types, an intriguing and possibly underestimated level of regulation. Still, it is important to notice that all studies reported so far in mammalian cells were conducted using cancer cell lines, which may not perfectly reflect the modification profile under physiological conditions. Noteworthy, the repertoire of tRNAs modified at position 9 observed in our study and by others (32) also largely overlaps with the targets reported for the yeast TRMT10A orthologue Trm10p (11).

tRNAs are a class of structurally and sequence related molecules, which pose a particular challenge to maturation enzymes that need to recognize a wide array of diverse substrates, yet exert sufficient specificity to prevent promiscuous activity. Inspecting the list of tRNAs methylated at position 9 in HAP1 cells, we could not identify any key feature distinguishing these tRNAs from those that are not methylated, in terms of the domains’ length or sequence composition. The only peculiarity that we observed was the consistently short, four or five nucleotides, variable loop in methylated tRNAs, possibly suggesting that a long variable loop may interfere with substrate binding; however, not all tRNAs with a short variable loop are methylated at position 9. Furthermore, the identity of the purine at position 9 is not the discriminating factor, since neither all tRNAs that have G9 are methylated *in vivo* by TRMT10A, nor all tRNAs that have A9 are methylated by TRMT10B; in fact, tRNA^Asp GTC 2^ appears to be the only substrate for TRMT10B. Possibly, additional specificity determinants may be in place for tRNA selection and methylation *in vivo* by the members of the TRM10 family, such as further modifications and/or RNA binding proteins. Interestingly, methyltransferases of the TRM10 family show a rather high degree of promiscuity *in vitro*, since they methylate a broad range of tRNAs that they do not methylate *in vivo* (10,11,14). Moreover, the overexpression of Trm10p in yeast leads to the methylation of additional tRNAs that normally are not methylated (11). During the revision of this manuscript others showed that, *in vitro*, TRMT10A and TRMT10B bind tRNAs that they do not methylate (13). Thus, the contribution of the binding affinity in determining which tRNAs are methylated at position 9 by the enzymes of the TRM10 family remains to be clarified.

Here, we show that TRMT10A and TRMT10B have a strict specificity for guanosine and adenosine, respectively, both *in vivo* and *in vitro*. Thereby, we have identified TRMT10B as the first adenosine-specific member of the TRM10 family in Eukarya. Remarkably, the mitochondrial form of the enzyme, consisting of the TRMT10C–SDR5C1 complex, is able to methylate both adenosine and guanosine (10), whereas in Archaea, orthologous methyltransferases were reported to be able to methylate either adenosine only, or both adenosine and guanosine (37). The unparalleled diversity of purine specificity in the TRM10 family is surprising considering that the *N*^1^ of guanosine is protonated at physiological pH, while the *N*^1^ of adenosine is not. We previously proposed an enzymatic mechanism involving a conserved aspartate in the catalytic centre, corresponding to D210 in human TRMT10A and D235 in human TRMT10B (10). In Archaeal homologs, a second aspartate residue was proposed to be specifically required for adenosine methylation (38); however, according to sequence alignment, this second aspartate is not conserved in human TRMT10B or TRMT10C. These observations suggest that the different specificities in the TRM10 family might have evolved independently multiple times in the different lineages, and that the purine specificity is apparently able to switch ‘easily’ during evolution. Future structural and biochemical studies shall clarify the enzymatic mechanism of the members of the TRM10 family of methyltransferases.

Trm10p, like many other tRNA modification enzymes, was shown to be not essential in yeast (8). Likewise, here we show that the KO of *TRMT10A* and/or *TRMT10B* does not cause any major growth phenotype in a human cell line. We only observed a slight tendency to slower growth in *TRMT10A*-KO cells; however, it shall be noted that due to the clonal selection of the KO cell lines, a bias toward the selection of faster-growing lines during clone isolation may not be excluded. In humans, loss-of-function mutations in *TRMT10A* are associated with a severe neurodevelopmental disorder and early-onset diabetes (15–18). A recent report showed that the depletion of TRMT10A in pancreatic β-cell lines causes an accumulation of 5′ fragments of tRNA^Gln^, which was suggested to induce apoptotic cell death (12). However, we did not observe fragmentation of tRNA^Gln^ or other tRNAs upon TRMT10A depletion in our cell system, indicating that tRNA fragmentation does not seem to be a generalized consequence of position 9 hypomodification. We observed a reduction in the steady-state levels of tRNA^iMet-CAT^ but not of tRNA^Asp-GTC^. The reduction might be due to turnover of tRNAs recognized as aberrant because of their destabilized structure and incorrect folding, or directly because of the lack of m^1^G9, similarly to what was shown in yeast, where the lack of m^1^A58 causes the selective degradation of tRNA^iMet-CAT^ (39). Still, downstream compensatory mechanisms, which may lead to transcriptional changes, cannot be excluded.

While many tRNAs are modified to m^2^_2_G at position 26 (4), initiator tRNA^iMet-CAT^ is characterized by a monomethylated m^2^G26, as well as m^2^G10 (33) (Supplementary Figure S6A), and no evidence for m^2^_2_G26 has been reported (32,40). Upon loss of position-9 methylation, we detected an increase in m^2^G content in tRNA^iMet-CAT^. The original study of tRNA^iMet-CAT^ isolated from human placenta showed that this tRNA is nearly fully modified at position 10 and only little modified at position 26. However, the extent of modification in tRNAs derived from other sources was never investigated, and it may vary depending on cell type. Due to the lack of sequence-context information in our MS method, we were unable to assign the increase of m^2^G to a specific position. RNA-SCRATCh experiments on G10 were not successful, due to the interference of G9 methylation on annealing of the ssODN guiding RNase H cleavage, while RT-based methods like NGS or primer extension are not able to detect m^2^G methylation. However, we showed that G26 is only partially methylated and unchanged upon the KO of *TRMT10A* or *TRMT10B*, suggesting that indeed G10 is likely the site of increased m^2^G methylation in tRNA^iMet-CAT^ upon the KO of *TRMT10A*. In the canonical tRNA structure, G10 and G26 are stacked on top of each other (Supplementary Figure S6B), and are methylated by TRM11/TRM112 and TRM1, respectively (41,42). G10 and G26 are located at the base of the D-stem and of the anticodon-stem, adjacent to G9 in the core of the L-shaped tRNA structure. It is intriguing to speculate that upon the lack of position-9 methylation, the hypomodified, structurally less stable tRNA may be recognized and methylated by other modification enzymes, in a compensatory fashion.

Noteworthy, according to our results, all copies of the human initiator tRNA are modified with m^1^G9, thus a dysfunction of this tRNA can potentially affect all cytosolic protein synthesis. It remains to be explained why the detrimental consequences of the loss of TRMT10A in the patients appear to be restricted to pancreas and nervous system. To date, over a hundred associations between RNA modification enzymes and human disease have been reported, among which neurological diseases are conspicuously overrepresented (reviewed in 2). The spectrum of consequences resulting from a deficiency of RNA modifications can be attributed to the diverse impact that modifications can have on cellular homeostasis. The loss of modifications in the anticodon loop of tRNAs or in the active centre of rRNAs can be detrimental for overall protein synthesis, affecting translation fidelity and efficiency (43); conversely, the lack of modifications that contribute to folding and stability can be well tolerated, and may exacerbate only in combination with metabolic or folding stress. In our KO cell lines, we did not observe any significant change in global protein synthesis, which is not entirely surprising considering that we did not observe any major growth phenotype. Remarkably, others previously reported even a minor increase in protein synthesis in TRMT10A-deficient cells (16), suggesting the presence of yet-to-be-clarified compensatory mechanisms. In this perspective, the lack of position 9 modification might cause suboptimal protein synthesis, and become deleterious only in certain cell types or developmental stages that require sustained massive protein synthesis, or upon intense bursts of translation in response to stimuli. Only further studies in physiologically relevant models will possibly shed light on the pathogenesis resulting from the lack of methylation at position 9 of tRNAs.

## DATA AVAILABILITY

All sequencing data were deposited at the European Nucleotide Archive (ENA) and are available under the accession number PRJEB31385.

## Supplementary Material

gkaa353_Supplemental_Files
