# Supplementary material for: Functional characterization of the human tRNA methyltransferases TRMT10A and TRMT10B
Source: Nucleic Acids Res. 2020 May 11;48(11):6157–69. doi: 10.1093/nar/gkaa353 (PMC7293042; doi:10.1093/nar/gkaa353)
Supplement: gkaa353_Supplemental_Files [file gkaa353_supplemental_files.zip › Vilardo Supplementary information.pdf]

Supplementary information

Functional characterization of the human tRNA methyltransferases TRMT10A and TRMT10B

Elisa Vilardo[1*], Fabian Amman[2], Ursula Toth[1], Annika Kotter[3], Mark Helm[3], and Walter Rossmanith[1*]

[1]Center for Anatomy & Cell Biology, Medical University of Vienna, 1090 Vienna, Austria

[2]Department of Theoretical Chemistry, University of Vienna, 1090 Vienna, Austria.

[3]Institute for Pharmacy and Biochemistry, Johannes Gutenberg-University, 55128 Mainz, Germany.

[*]To whom correspondence should be addressed. Tel: +43 1 40160 37724; Email: elisa.vilardo@meduniwien.ac.at. Correspondence may also be addressed to Tel. +43 1 40160 37512 Email: walter.rossmanith@meduniwien.ac.at.

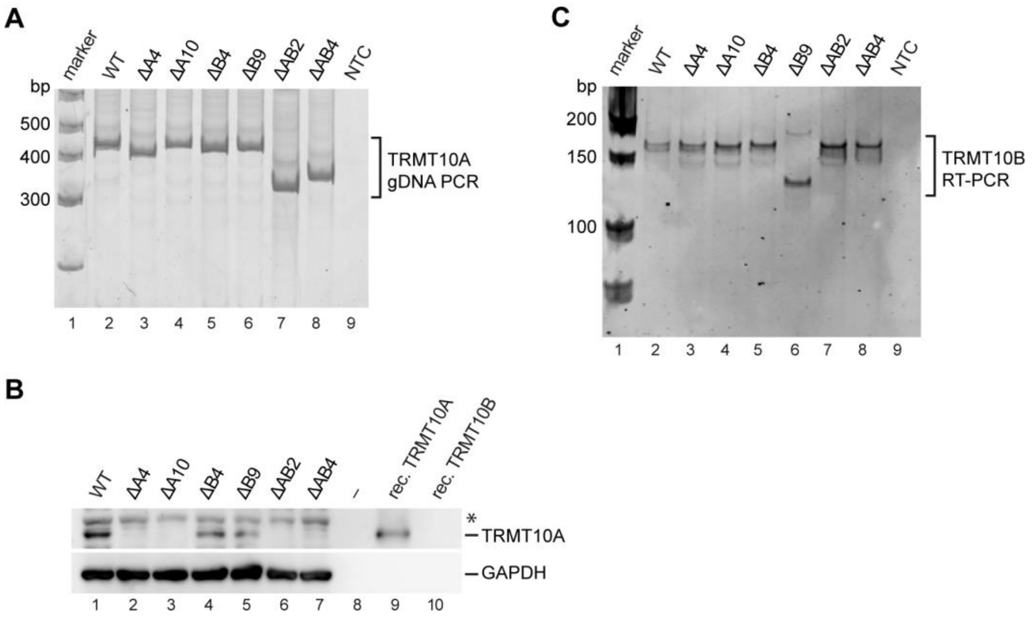

Supplementary Figure 1 Characterization of HAP1 KO clones. (**A**) PCR amplification of the gene of TRMT10A in the region targeted by CRISPR/Cas genome editing. DNA amplicons were separated by 8% PAGE and stained with GelRed. The molecular weight of selected marker bands is indicated to the left. (**B**) Western blot analysis of TRMT10A in WT HAP1 and KO clones. Recombinant TRMT10A and TRMT10B were loaded along as controls. GAPDH was used as loading control. An additional unspecific signal visible in the blot is labelled with an asterisk. (**C**) RT-PCR analysis of the TRMT10B-mRNA region targeted by CRISPR/Cas genome editing. DNA amplicons were separated by 8% PAGE and stained with GelRed. The molecular weight of selected marker bands is indicated to the left. The multiplet appearance of the bands was likely due to a PAGE artefact, as visible also in the marker lane.

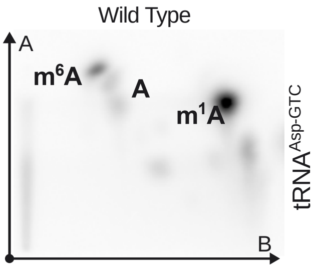

Supplementary Figure 2 Analysis of position 9 methylation of $tRNA^{Asp\text{-}GTC}$. RNA-SCRATCh of $tRNA^{Asp\text{-}GTC}$ from the wild type HAP1 sample shown in (Figure 4D) was resolved by two-dimensional TLC. The arrows indicate the direction of migration of the first (A) and the second (B) solvent. The identity of the spots is labelled within the plate. The small amount of m^6A visible in the TLC apparently resulted from Dimroth rearrangement of m^1A during sample preparation (1). Weak background spots corresponding to different nucleosides monophosphates and/or their modified forms are visible; they are likely due to purification contaminants or partial degradation before radioactive labelling.

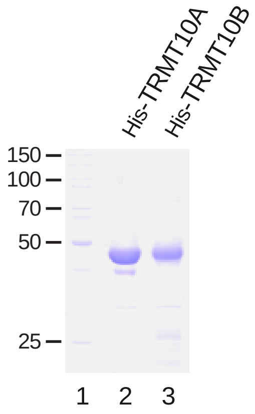

Supplementary Figure 3 Recombinant TRMT10A and TRMT10B used in this study. 5.8 µg of TRMT10A and 5 µg of TRMT10B were resolved by SDS-PAGE and stained with Coomassie brilliant blue. The molecular weight of selected marker proteins (lane 1) is indicated to the left.

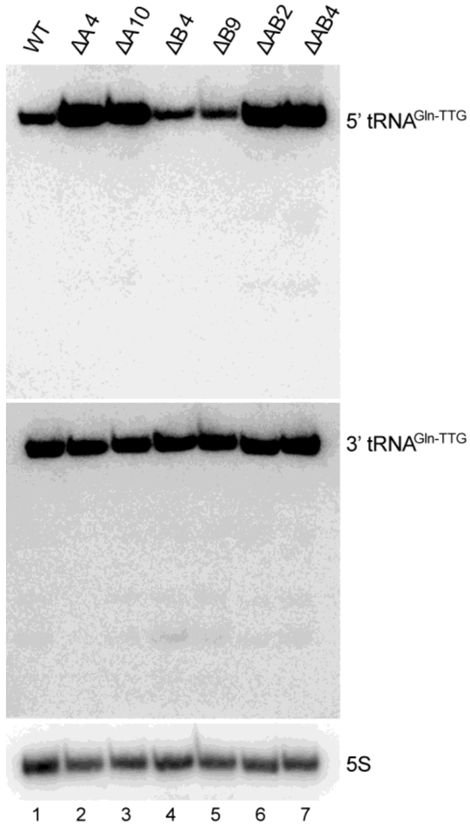

Supplementary Figure 4 Analysis of tRNA$^{Gln\text{-}TTG}$ integrity upon the KO of TRMT10A and/or TRMT10B. tRNA$^{Gln\text{-}TTG}$ was analysed by northern blotting with a probe against its 5' end (upper panel) or its 3' end (middle panel). This is the same blot as shown in Figure 6A, but enhanced to see low intensity signals. Moreover, a larger section including the lower molecular weight range is shown here, to specifically screen for tRNA fragmentation upon the KO of TRMT10A. 5S rRNA is shown as loading control (bottom panel, same shown in Figure 6A).

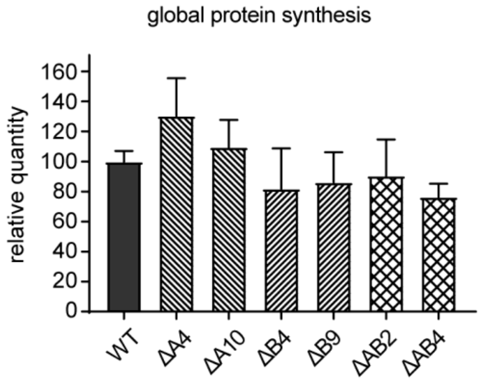

Supplementary Figure 5 Global protein synthesis assay of HAP1 cells and the derived TRMT10A and/or TRMT10B KO clones. Protein synthesis was measured by incorporation of OP-puro and fluorescent labelling, followed by flow cytometry. Median cellular fluorescence intensity was normalized to that of wild type cells. The data are shown as mean and SD of six replicates, relative to wild type (N=6).

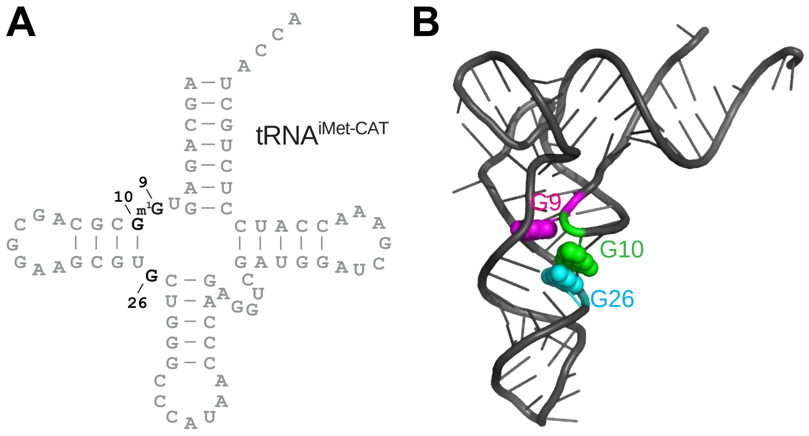

Supplementary Figure 6 tRNA structure and relevant modification sites. (**A**) Canonical cloverleaf representation of human tRNA$^{\text{iMet-CAT}}$. Methylation of position 9, and positions G10 and G26 are highlighted in black. (**B**) Cartoon display of the structure of *E. coli* tRNA$^{\text{iMet-CAT}}$ (PDB: 3CW5). The guanosines at position 9, 10, and 26 are displayed as spheres and labelled; structure display generated with PyMOL 2.0.7 software (2).

Supplementary reference

1. Jones, J.W. and Robins, R.K. (1963) Purine nucleosides. III. Methylation studies of certain naturally occurring purine nucleosides. Journal of the American Chemical Society, 85, 193-201.

2. Schrodinger, L.L.C. (2015).
